# Enhancing Well-Being and Social Connectedness for Māori Elders Through a Peer Education (Tuakana-Teina) Programme: A Cross-Sectional Baseline Study

**DOI:** 10.3389/fpubh.2021.775545

**Published:** 2021-12-08

**Authors:** John G. Oetzel, Stacey Ruru, Yingsha Zhang, Mary Louisa Simpson, Sophie Nock, Pare Meha, Kath Holmes, Marama Clark, Hariata Adams, Ngapera Akapita, Kawarau Ngaia, Shane Murphy, Reuben Moses, Rangimahora Reddy, Brendan Hokowhitu

**Affiliations:** ^1^Waikato Management School, University of Waikato, Hamilton, New Zealand; ^2^Faculty of Māori and Indigenous Studies, University of Waikato, Hamilton, New Zealand; ^3^Rauawaawa Kaumātua Charitable Trust, Hamilton, New Zealand; ^4^Poutiri Trust, Te Puke, New Zealand; ^5^Te Korowai Hauora o Hauraki, Thames, New Zealand; ^6^Te Korowai o Ngāruahine, Hāwera, New Zealand; ^7^Te Roopu Tautoko ki te Tonga, Dunedin, New Zealand; ^8^University of Queensland, Brisbane, QC, Australia

**Keywords:** health equity, older Māori health, peer education, Indigenous ageing, social support, health-related quality of life

## Abstract

**Background:** Māori kaumātua (elders) face stark health and social inequities compared to non-Māori New Zealanders. The tuakana-teina (older sibling-younger sibling) peer education programme is a strengths-based approach to enhance well-being and social connectedness. The purpose of this study is to present the baseline data from this programme and identify correlates of well-being outcomes.

**Method:** Participants included 128 kaumātua who completed a self-report survey about health-related quality of life, spirituality, social connection and loneliness, life satisfaction, cultural identity and connection, elder abuse, health service utilisation and demographics.

**Findings:** Multiple regression models illustrated the following correlates of outcomes: (a) self-rated health: needing more help with daily tasks (β = −0.36) and housing problems (β = –0.17); (b) health-related quality of life: needing more help with daily tasks (β = –0.31), housing problems (β = –0.21), and perceived autonomy (β = 0.19); (c) spiritual well-being: understanding of tikanga (cultural protocols) (β = 0.32) and perceived autonomy (β = 0.23); (d) life satisfaction: social support (β = 0.23), sense of purpose (β = 0.23), cultural identity (β = 0.24), trouble paying bills (β = –0.16), and housing problems (β = –0.16); (e) loneliness: elder abuse (β = 0.27), social support (β = –0.21), and missing pleasure of being with whānau (extended family) (β = 0.19).

**Conclusions:** Key correlates for outcomes centred on social support, housing problems, cultural connection and perceived autonomy. These correlates are largely addressed through the programme where tuakana/peer educators provide support and links to social and health services to teina/peer recipients in need. This study illustrates needs and challenges for kaumātua, whilst the larger programme represents a strengths-based and culturally-centred approach to address health issues related to ageing in an Indigenous population.

## Introduction

The Aotearoa (New Zealand) population is ageing and there are a number of health and social challenges that can result including loneliness and social isolation, chronic conditions and end-of-life issues (1). There are also many positive features from ageing and there is a focus on ageing well in the research space in Aotearoa (2). The stark health and social inequities between Māori (Indigenous people of Aotearoa) kaumātua (elders) and non-Māori elders (3–5) are of key importance for this study. For example, Russell and colleagues (6) highlighted that “Māori experience systematic disparities in health outcomes, determinants of health, health system responsiveness, and representation in the health sector workforce” (p. 10). These inequities are due to unequal distribution of social determinants (e.g., income, education, housing), lack of access to social and/or health services and structural discrimination resulting from the effects of colonisation (3, 7, 8).

Māori comprise about 17% of the total population and about 7% of Māori are 65 or older (9). Growing up in the 1940s, 50s and 60s, many of today's kaumātua experienced a significantly more racist society than present including marginalisation, socio-economic disadvantage, government policies that fostered monoculturalism and an education system that discouraged or even punished people for speaking te reo Māori (Māori language) and/or for practising tikanga Māori (Māori cultural protocols) (10, 11). A further example of such practises is the Tohunga (traditional healer) Suppression Act, which banned the use of traditional healers and medicines in an effort to assimilate Māori into the British health system and was not repealed until 1962 (12). Indeed, there is a growing literature that not only foregrounds the effects of colonisation in relation to Indigenous health inequities, it also assumes a causality between what is now increasingly referred to as colonial “historical trauma” and epistemological violence. The research, thus, recognises that kaumātua may have vastly different experiences of tikanga Māori and te reo Māori, with many having varying degrees of cultural dissonance and feelings of separation and even fear of interaction with their own Indigenous culture. Such cultural dissonance has led to a number of health and social inequities and poor health outcomes (13–15). Moreover, kaumātua have generally faced a dominant society that has failed to realise their full potential as they age, even whilst Māori culture (in abstraction at least) upholds elders as, “carriers of culture, anchors for families, models for lifestyle, bridges to the future, guardians of heritage, and role models for younger generations” (p. 14) (16).

Whilst there are some interventions to address health inequities, many are based on a deficit approach (4, 7, 17). A deficit-based research approach focuses on marginalised groups as “problems” to be compared with dominant groups as a baseline and “fixed” via interventions. In contrast, our strength-based approach emphasises the predominance of a Māori worldview of health and social relations, with a focus on tikanga to guide solutions and conduct research. In some cases, the deficit model implicitly and explicitly blames Māori for the deficit rather than examining systemic and structural issues including loss of cultural connection resulting from colonisation and marginalisation (18, 19). The current study involves a strengths-based approach that foregrounds kaumātua mana motuhake, or the desire to achieve actualization through independence and autonomy at an individual and collective level (20) and tuakana-teina as a fundamental cultural concept.

The tuakana-teina (literally older sibling-younger sibling; peer educator and peer recipient in this study) peer education model was developed in partnership between a kaumātua health and social service provider and university researchers (21). The purpose of the model was to use the experience and resources that the tuakana (kaumātua with communication skills and life experience) have to assist teina (kaumātua in need of support, guidance, etc.) in dealing with various life transitions to enhance overall hauora (health) and linking those kaumātua in need to health and social services. The initial implementation of the model demonstrated that kaumātua provided strong peer education skills and resources and that there were benefits to health-related quality of life, social support, and cultural connection for participants of this peer education model (22, 23). The success of this initial implementation led to the expansion of the model to be delivered in five additional Māori health and social service providers as part of the Kaumātua Mana Motuhake Poi (KMMP) programme of research funded by the Ageing Well National Science Challenge in Aotearoa (https://www.ageingwellchallenge.co.nz/) (20).

The purpose of the current study is 2-fold. The first purpose is to present the baseline data from the tuakana-teina peer education model to establish psychometric characteristics and the initial comparison point for the key measures. The second purpose is to illustrate the correlates of key health outcomes relative to the te whare tapa whā model of health (four walls of the community meeting house) (13, 24). Te whare tapa whā is one of a number of Māori models of health that emphasises a holistic perspective of health; it was chosen by the partnership and advisory boards guiding this project. The four elements include the following: te taha whānau (social health), te taha hinengaro (psychological/mental health), te taha wairua (spiritual health), and te taha tinana (physical health). Examining the correlates provides indications of elements important to these key outcomes, which can provide insight for researchers and practitioners and can also reinforce whether the peer education model is focused on key attributes.

## Method

The tuakana-teina peer educator model (20) involves kaumātua (tuakana) serving as peer educators for other kaumātua (teina). The design of the larger study is a mixed-methods, pre-test and two post-test, staggered design with three providers receiving the approach first and then two receiving it on a delayed basis. The larger project is guided by the He Pikinga Waiora Implementation Framework (25). This framework centres Kaupapa Māori (17, 26), which provides a culturally appropriate methodology as it normalises Māori worldviews and practises (27); it also focuses on self-determination and the validity of a Māori epistemology (28). The framework also emphases culture-centredness, community engagement, systems thinking and integrated knowledge translation. Overall, the framework is a participatory approach recognising the unique strengths that each partner brings (29) along with seeking ways to sustain and integrate the intervention within the larger social and health system (30). The research team involves a partnership of a number of Māori health and social service providers and university researchers. The providers worked with the original partnership to adapt elements of the tuakana-teina model to fit their local community and this process is described elsewhere (20). The project is registered with the Australia New Zealand Clinical Trial Registry (ACTRN12620000316909).

The current study is a cross-sectional survey to establish baseline attributes of key outcomes for the larger study. In the larger project, participants complete a survey questionnaire at three different time periods and the current study focuses on the first or baseline questionnaire, which was completed at the initial point of consent for the study. Participants completed the self-report survey questionnaire about such constructs as health-related quality of life, spirituality, social connection and loneliness, life satisfaction, cultural identity, elder abuse, health service utilisation, and demographics.

### Participants

Participants included kaumātua from five different Māori health and social service providers in five different regions in Aotearoa. Each provider was originally asked to select four tuakana based on effective peer educator attributes for ageing research (31) and attributes within Te Ao Māori such as mana (status), pono (honesty), aroha (love, compassion), and tika (fairness) (16, 32). Each tuakana serves as a peer educator for six teina. Providers were asked to provide a list of eligible kaumātua for recruitment based on what the providers decided were greatest needs and then the list was randomised with a target of recruiting 24 teina. Greatest needs included an array of features including loneliness, health needs and social needs. The eligibility criteria were flexible so providers could include kaumātua deemed to need services. Exclusion criteria were being too unwell to participate such as kaumātua with dementia.

### Measures

We included a number of measures to address our core themes of hauora and mana motuhake. Validated scales from previous empirical research (21, 33) include the following for hauora [hinengaro (mental), tinana (physical), wairua (spiritual), and whanaunga (social)]: self-reported health (34, 35), mental/physical health-related quality of life (HRQOL) (36, 37), spirituality (38), cultural connection (39), loneliness [one item from Hayman et al. (1) and three items from Waldegrave et al. (40)], historical trauma [four items created for this study although modelled off Whitbeck et al. (41)] and perceived and desired social support (42). For mana motuhake, we included perceived autonomy (43), life satisfaction (44), sense of purpose (45), and economic well-being (38). Further additional constructs were identified by community providers and researchers as relevant to the project: elder abuse (46), health service utilisation and knowledge [one item from Oetzel et al. (22) and two items created for this study] and housing problems (38). These constructs were measured by three different types of scales: (1) Likert-type scales ranging from 3 to 6 points; (2) 11-point semantic differential scales; and (3) category scales.

There was a total of 42 items in the survey. The participants were invited to complete the questionnaire on their own or via a structured interview administered by a Māori community researcher and could have a support person present if they desired. The majority of questionnaires were completed at the provider's location with a few completed in participants' homes. The questionnaire includes both Māori and English language versions. The Māori language version involved a translation and back-translation procedure to ensure equivalence to the English version. The questionnaire was written with plenty of spacing and large font to support the reading needs of kaumātua. Participants received a $50 gift card. Ethical approval was provided by the University of Waikato's Human Research Ethics Committee, HREC (Health) 2019#81. Further cultural safety and protection of mana motuhake were protected by using a culturally-appropriate approach for data collection developed in previous projects (47, 48).

### Data Analysis

The questionnaires were entered into SPSS for the data analysis. The reliability of scales was assessed with Cronbach's alpha. Low scale reliability for the loneliness and social support scales resulted in using principal components factor analysis with varimax rotation to identify key factors. Criteria for inclusion was a primary factor loading of 0.6 and a secondary loading at least 0.2 lower than the primary loading. Descriptive statistics of items, including bivariate correlations were provided. Five multiple linear regression models were run to identify the correlates of five key outcomes variables: self-rated health, HRQOL, spiritual wellbeing, loneliness, and life satisfaction; the first four correspond to health and the latter to mana motuhake. The independent variables were the remaining items/scales that had a statistically significant bivariate correlation with the outcomes. The forward method was used to build the multiple regression model.

## Results

**Figure 1:**
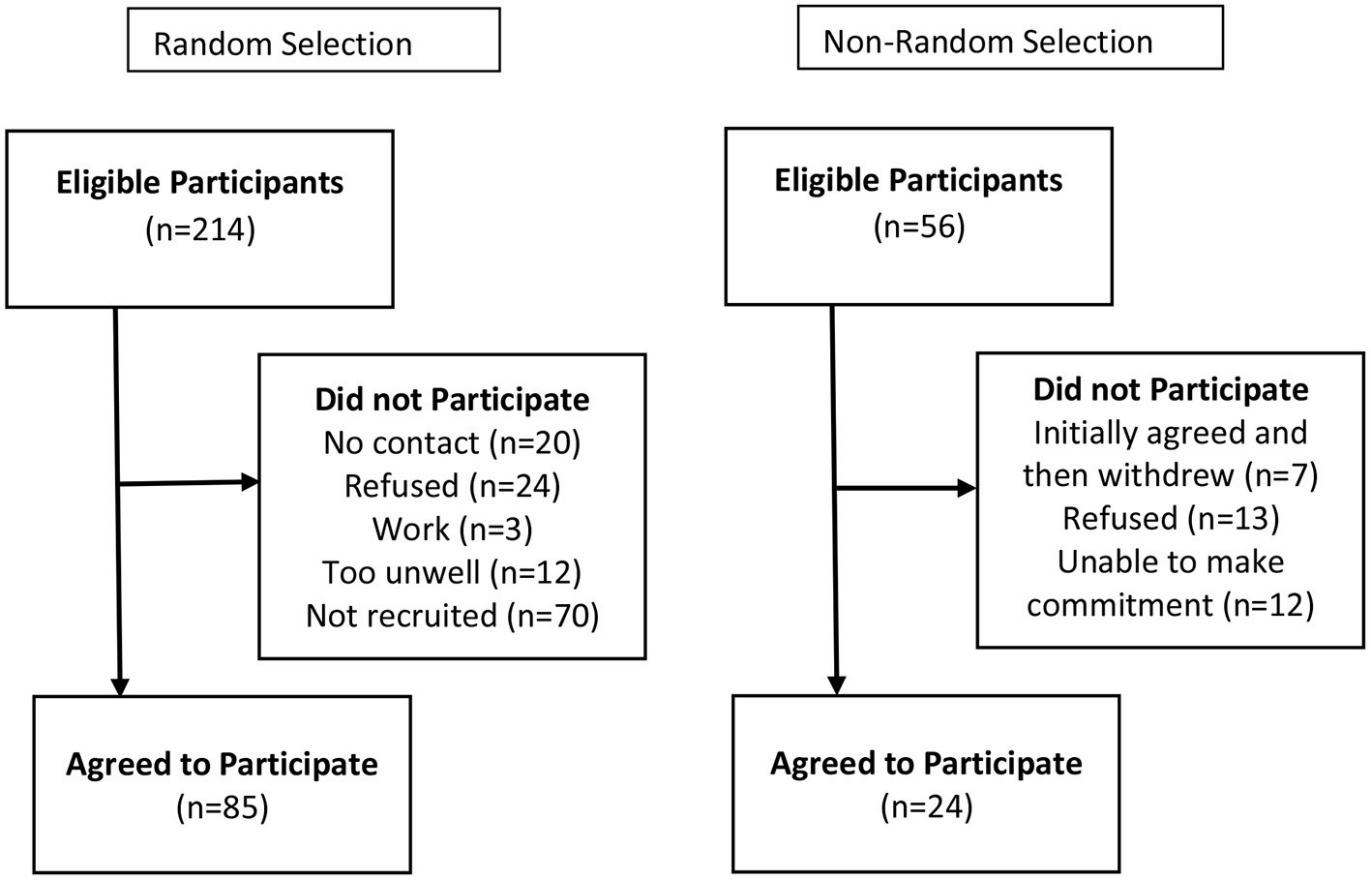
Recruitment of Teina from random and non-random selection.

A total of 128 kaumātua participated in the baseline survey. One of the providers was not able to provide an initial list and instead recruited participants at community meetings through a non-random method (4 tuakana and 24 teina). Specifically, this provider attended the meetings and asked for the contact information of potential participants and then contacted individuals directly. The other four providers included the following: (a) 5 tuakana and 25 teina; (b) 4 tuakana and 24 teina; (c) 6 tuakana and 21 teina; and (d) 15 teina. The extra three tuakana and one teina in the first three providers included participants who withdrew due to health reasons after completing the baseline survey and prior to the start of the programme. The final provider was not able to meet the final target due to refusals; further, three tuakana did not complete the baseline survey as they were recruited after its administration. There were 97 women and 31 men with an average age of 71.31 (*SD* = 8.87). All participants were Māori. Figure 1 provides a summary of the recruitment numbers.

Factor analysis revealed two factors for social support and loneliness items. However, only one factor of social support (four items) had sufficient reliability and thus the remaining were used as single items for constructs. Table 1 displays the mean, standard deviation, bivariate correlations and Cronbach's alpha for each scale. The table also displays the response scale for the items or scales. Self-rated health, HRQOL, spiritual well-being and health service utilisation/knowledge were scored by anchoring high scores in the response scale to 100 and low scores to 0 with equivalent space in between the remaining responses. This follows the RAND approach to the quality of life scores (49).

**Table 1:** Descriptive statistics and bivariate correlations.

**Construct**	***M***	***SD***	**Self-rated health**	**HRQOL**	**Spiritual wellbeing**	**Life satisfaction**	**Loneliness**	**Support**	**Elder abuse**	**Cultural identity**	**Sense of purpose**	**Health service**	**Housing Problems**	**Historical trauma**	**Understanding of tikanga**	**Missing pleasure with whānau**	**Needing more Help with Daily Tasks**	**Needing more emotional support**	**Perceived autonomy**	**Trouble Paying Bills**
Self-rated Health-1 item (0–100)	64.73	23.34	n/a																	
HRQOL-7 items (0–100)	66.80	19.92	0.72^**^	0.88																
Spiritual Wellbeing-1 item (0–100)	77.97	18.41	0.39^**^	0.41^**^	n/a															
Life Satisfaction-1 item (0–10)	8.42	1.80	0.36^**^	0.37^**^	0.36^**^	n/a														
Loneliness -1 item (1–4)	1.57	0.64	−0.18^*^	−0.23^*^	−0.05	−0.22^*^	n/a													
Support-4 items (1–5)	3.66	0.67	0.02	0.12	0.18^*^	0.36^**^	−28^**^	0.69												
Elder Abuse-4 items (0–4)	0.15	0.26	−0.13	−0.19^*^	−0.13	−0.16	0.34^**^	−0.18^*^	0.71											
Cultural Identity-2 items (1–5)	3.90	0.94	0.10	0.12	0.26^**^	0.34^**^	−0.13	0.23^**^	−0.23^**^	0.71										
Sense of Purpose-3 items (1–5)	3.93	0.71	0.18^*^	0.10	0.16	0.32^**^	−0.13	0.10	−0.04	0.13	0.84									
Health Service Use and Knowledge -3 items (0–100)	69.62	22.43	0.05	−0.01	0.06	0.14	0.07	0.14	−0.17	0.12	0.07	0.65								
Housing Problems-3 items (1–4)	1.60	0.67	−0.21^*^	−0.29^**^	−0.21^*^	−0.33^**^	0.19^*^	−0.17	0.21^*^	−0.11	−0.22^*^	−0.06	0.65							
Historical trauma -4 items (1–5)	2.61	1.20	0.08	0.09	−0.05	0.15	−0.02	−0.06	0.08	−0.03	0.05	−0.12	−0.02	0.87						
Understanding of Tikanga (cultural protocols) -1 item (1–4)	3.36	0.79	0.12	0.13	0.39^**^	0.33^**^	−22^**^	0.17	−0.27^**^	0.39^**^	0.26^**^	0.12	−0.13	−0.07	n/a					
Missing Pleasure of being with Whānau-1 item (1–5)	3.51	1.14	0.03	0.02	−0.05	0.07	0.26^**^	−0.11	0.16	0.17	−0.03	0.08	0.05	0.03	−0.03	n/a				
Needing More Help with Daily Tasks-1 item (1–4)	1.87	0.82	−0.38^**^	−0.32^**^	−0.05	−0.10	0.15	0.03	0.07	0.08	−0.04	0.03	0.11	0.09	<0.01	0.24^**^	n/a			
Needing More Emotional Support-1 item (1–4)	1.86	0.79	−0.17	−0.18^*^	−0.07	−0.16	0.13	−0.12	0.16	−0.04	−0.09	−0.18^*^	0.14	−0.08	−0.06	0.16	0.27^**^	n/a		
Perceived Autonomy-1 item (0–10)	9.24	1.47	0.15	0.23^**^	0.33^**^	0.28^**^	−0.18^*^	0.14	−0.32^**^	0.22^*^	0.31^**^	<0.01	−0.28^**^	−0.18^*^	0.32^**^	0.05	0.06	−0.10	n/a	
Trouble Paying Bills-1 item (1–3)	1.34	0.71	−0.10	−0.21^*^	−0.12	−0.23^**^	0.04	−0.12	0.14	0.01	0.02	−0.03	0.30^**^	0.04	−0.04	0.04	< −0.01	0.09	−0.24^**^	n/a

*Higher mean values reflect higher values of construct*.

* *p < 0.05*;

** *p < 0.01; Cronbach's alpha listed on the diagonal*.

Table 2 displays the significant correlates for the five outcome variables. The multiple regression model for self-rated health revealed two significant correlates [*F*_(2,125)_ = 13.07, *p* < 0.001, adj *R*^2^ = 0.16]. Needing more help with daily tasks and housing problems negatively correlated with self-rated health. The multiple regression model for HRQOL was significant [*F*_(3,123)_ = 10.17, *p* < *0.0*01, adj *R*^2^ = 0.18]. Perceived autonomy was a positive correlate of HRQOL; needing more help with daily tasks and housing problems were negative correlates of HRQOL. The multiple regression model for spiritual well-being was significant [*F*_(2,125)_ = 15.69, *p* < 0.001], adj *R*^2^ = 0.19. Understanding of tikanga and perceived autonomy were positive correlates of spiritual well-being. The model for life satisfaction was significant [*F*_(5,122)_ = 11.84, *p* < 0.001, adj *R*^2^ = 0.30]. Life satisfaction was positively related with support, sense of purpose and cultural identity; it was negatively related with trouble paying bills and housing problems. Finally, the model for loneliness was significant [*F*_(3,123)_ = 10.30, *p* < 0.001, adj *R*^2^ = 0.18]. Loneliness was negatively associated with support and positively associated with elder abuse and missing pleasure with whānau (extended family).

**Table 2:** Multiple regression models for key health-related outcomes.

**Correlates**	***B***	***SE B***	**β**	***p***
**Self-rated health**				
Need more help with daily tasks	−10.33	2.34	−0.36	<0.001
Housing problems	−5.97	2.87	−0.17	0.039
**HRQOL**				
Need more help with daily tasks	−7.41	1.98	−0.31	<0.001
House problem	−6.18	2.53	−0.21	0.016
Perceived autonomy	2.49	1.14	0.19	0.031
**Spiritual Well-being**				
Understanding of Tikanga	7.40	1.96	0.32	<0.001
Perceived autonomy	2.90	1.05	0.23	0.007
**Life satisfaction**				
Support	0.61	0.21	0.23	0.004
Sense of purpose	0.58	0.20	0.23	0.004
Cultural identity	0.46	0.15	0.24	0.002
Trouble paying bills	−0.42	0.20	−0.16	0.039
House problem	−0.44	0.22	−0.16	0.048
**Loneliness**				
Elder abuse	0.65	0.20	0.27	0.001
Support	−0.20	0.08	−0.21	0.011
Miss pleasure with Whāna	0.11	0.05	0.19	0.022

## Discussion

The purpose of this study was to provide the baseline attributes and correlates for key health and social outcomes for the tuakana-teina peer education programme. Descriptive statistics indicated good health-related quality of life, high spiritual well-being and life satisfaction, moderate levels of social support and health service utilisation, and low levels of elder abuse and loneliness. Whilst there are not direct comparisons to other populations in Aotearoa on these scales, the descriptive statistics do support relatively strong characteristics in this sample and the importance of not presuming deficits or taking a deficit approach (18, 19). Further, the responses illustrate that there is room for improvement on most of the scales and items considered in the study so the tuakana-teina peer education model can have a positive impact on these variables (22).

Social support (the scale and individual items) was a significant correlate for four of the five outcomes (all except spiritual well-being). Social support has long been found to be an important correlate for a variety of health and well-being outcomes for Indigenous and non-Indigenous populations (39, 50–54). In the current study, we considered aspects of tangible and emotional support, finding that needing more tangible support was negatively associated with HRQOL and self-rated health, whilst emotional support was positively associated with life satisfaction and negatively associated with loneliness. For older people, maintaining positive social connection and relationships is critical for life expectancy and quality of life (54, 55).

Perceived autonomy was an important correlate for two key outcomes (HRQOL and spiritual well-being). Perceived autonomy is a key component of mana motuhake and reflects the importance of collective and individual determination of one's life (18, 20, 56). Autonomy is a historical element in Aoteoroa in the relationships between the settlers and Indigenous people. Te Tiriti o Waitangi (The Treaty of Waitangi), the founding document of Aotearoa (a treaty based on trust signed in 1840 by Governor Hobson on behalf of the British Crown and a number of Māori chiefs), guaranteed Māori the right of self-determination in the Māori version and yet has historically not been allowed by the State (57, 58). This collective history is one aspect of self-determination, but certainly there is the desire for independence and autonomy as one ages as well (43).

Problems with current housing was negatively associated with self-rated health, HRQOL and life satisfaction. Having a stable and positive housing situation is important for older people who desire to age in place and to age well (59, 60). These factors are particularly important for people who are retired and living on a pension or other retirement income (60). This is a key element in Aotearoa as Māori home ownership is lower than that of Pākehā [New Zealand European and there are escalating rental prices (61)]. Housing is often not thought of as a health problem, but mould or poor heating have direct impacts on physical and mental health. Further, the financial aspects of housing can negatively impact kaumātua health through not being able to afford healthcare and other related components. It is often why many Māori providers include housing aspects as part of an integrated care model (either directly or in partnership with housing providers).

Cultural connection as measured by cultural identity and knowledge of tikanga (cultural protocols) was positively correlated with spiritual well-being and life satisfaction. Kaumātua value their tikanga and seek ways to learn and practise it. Many of the providers offer cultural programmes to address cultural connectivity and learn te reo Māori (language). Cultural connectivity is an important element particularly for older Māori who come from a generation where State policy discouraged valuing their language and culture (15, 62). The cultural dissonance created by these policies has been shown to have a negative impact on health and social outcomes (15, 41, 63).

This study has several important implications for understanding Indigenous perspectives on ageing. Our partnership reinforces the importance of understanding health from a holistic perspective. During the process of developing the programme, our partners and kaumātua stated the need to examine social, cultural, spiritual, physical and mental aspects of health and well-being. Consistent with Māori models of health (24), ageing well for kaumātua includes a balanced perspective of multiple elements. Our measures reflect this holism by including specific scales and items for all of these elements.

Relatedly, this study supports the relevance and focus on mana motuhake and the context of a colonial history. Many of the current kaumātua were discouraged from, and punished for, speaking te reo Māori (the Māori language) in the education system (10). This creates a cultural dissonance that results from a hegemonic dominant culture subjugating an Indigenous culture. Mana motuhake emphasises the status, independence and autonomy of kaumātua identifying their own needs and solutions around health and well-being in order to encourage “ageing well.” Kaumātua are revered in Māori culture and afforded support for their needs and the respect to make their own decisions. The tuakana-teina peer education model was developed to support kaumātua mana motuhake by having kaumātua support and help each other. The current study illustrates the importance of emotional support, tangible support, sense of purpose and cultural identity for enhancing the mana motuhake and life satisfaction for kaumātua.

Further, the tuakana-teina programme addresses key correlates as identified in this study. The programme is designed to provide emotional support and cultural connections through peers. Conversations are culturally grounded to support learning and respect for tikanga. They are also guided to explore holistic needs around health and well-being and provide links to tangible resources to support these needs. A previous iteration and implementation of the model supports its effectiveness (22, 23) with the current study determining whether the benefits can be translated to other communities as well.

Despite the strengths of the study, there are some key limitations that should be noted. Firstly, all of the measures are self-reported, which may result in some perceptual bias, and some of the measures had lower than ideal reliability. During the co-design phase of the partnership, the providers emphasised the importance of kaumātua being able to describe their own well-being and that self-report measures would be the best way to reflect their mana motuhake. Nonetheless, there are limited measures that have been validated and shown to be reliable with the kaumātua population and hence this is an area in need of future research. Secondly, the aim of the project was to identify kaumātua with the greatest needs and yet the participants have relatively good to high scores on the key outcomes. The recruitment strategy of using Māori providers to identify the greatest needs was deemed appropriate during planning stages, but it appears that some kaumātua with needs may not be connected to these providers. Thirdly, we did have some non-random selection from one provider, which may introduce some bias in recruitment. However, the response patterns in this provider were largely consistent with other providers so this limitation is somewhat mitigated.

## Conclusion

In conclusion, this study provided some key psychometric details and baseline attributes of the key outcome variables for the tuakana-teina peer education model that is currently underway. This information provides the comparison point for later evaluation of the intervention. Additionally, the study identifies key correlates for outcomes organised around the te whare tapa whā model including social support, housing problems, cultural identity/understanding tikanga, and perceived autonomy. These correlates are largely addressed through the programme where tuakana/peer educators provide support and links to social and health services to teina/peer recipients in need. This study illustrates key needs and challenges for kaumātua, whilst the larger programme represents a strengths-based and culturally-centred approach to addressing ageing in an Indigenous population.

## Data Availability Statement

The raw data supporting the conclusions of this article will be made available by the authors, without undue reservation.

## Ethics Statement

The studies involving human participants were reviewed and approved by the project was approved by the Human Research Ethics Committee, University of Waikato (HREC2019#81). The patients/participants provided their written informed consent to participate in this study.

## Author Contributions

JO led conceptualisation and writing of the manuscript and served as Co-PI on project. SR completed the literature review and contributed to writing of the background. YZ completed data analysis and contributed to methods and results sections. MS led implementation of peer education programme and evaluation. SN and PM co-led implementation of peer education programme and evaluation. MC, HA, NA, KN, SM, and RM were primary contacts for participants and led data collection in their organisations. RR provided leadership to overall research programme and served as Co-PI of project. BH provided leadership to overall research programme and served as PI of project. All authors contributed to research design and data interpretation. They also reviewed, edited and approved the manuscript.

## Funding

The project was funded by the Ageing Well National Science Challenge, New Zealand's Ministry of Business, Innovation and Employment (18566SUB1953); Brendan Hokowhitu (PI), JO and RR (Co-PIs). The authors maintain sole responsibility for the research design, data collection, data analysis, and interpretation of the findings. The project underwent peer review by the funding body.

## Conflict of Interest

The authors declare that the research was conducted in the absence of any commercial or financial relationships that could be construed as a potential conflict of interest.

## Publisher's Note

All claims expressed in this article are solely those of the authors and do not necessarily represent those of their affiliated organizations, or those of the publisher, the editors and the reviewers. Any product that may be evaluated in this article, or claim that may be made by its manufacturer, is not guaranteed or endorsed by the publisher.
