# Supplementary material for: Enhancing Well-Being and Social Connectedness for Māori Elders Through a Peer Education (Tuakana-Teina) Programme: A Cross-Sectional Baseline Study
Source: Front Public Health. 2021 Dec 8;9:775545. doi: 10.3389/fpubh.2021.775545 (PMC8692656; doi:10.3389/fpubh.2021.775545)
Supplement: Supplementary file 1 [file Table_1.pdf]

Survey Items and Anchor Points in Response Scale

Self-rated Health
Overall, how would you rate your health during the past 4 weeks? (excellent-very poor)

Health-Related Quality of Life
During the past 4 weeks, how much have you been bothered by emotional problems (such as feeling anxious, depressed or irritable)? (not at all-extremely)

During the past 4 weeks, how much did personal or emotional problems keep you from doing your usual work, school or other daily activities? (not at all-could not do daily activities)

During the past 4 weeks, how much did physical health problems limit your physical activities (such as walking or climbing stairs)? (not at all-could not do physical activities)

During the past 4 weeks, how easy was it for you to do your daily work, both at home and away from home, with your physical health? (very easy-could not do daily work)

How much bodily pain have you had during the past 4 weeks? (none-very severe)

During the past 4 weeks, how much energy did you have? (very much-none)

During the past 4 weeks, how much did your physical health or emotional problems limit your usual social activities with family or friends? (not at all-could not do social activities)

Spiritual Wellbeing
How would you rate your overall spiritual wellbeing? (excellent-very poor)

Life Satisfaction
Please think about how you would rate your life overall with 0 = your worse possible life and 10 = to your best possible life. Where would you rate your life on this scale?

Loneliness
How often do you feel lonely? (always feel lonely-never feel lonely)

Social Support
I have really close whānau I can talk to. (strongly disagree-strongly agree)

I spend plenty of time with my whānau. (strongly disagree-strongly agree)

When you need extra help, can you count on whānau or others to help with daily tasks like grocery shopping, cooking, house cleaning, telephoning, giving you a ride? (always-never)*

Can you count on whānau or others to provide you with emotional support? (always-never)*

Elder Abuse
Do you feel uncomfortable with anyone in your whānau? (yes-no)

Has any of your whānau forced you to do things you didn’t want to do? (yes-no)

Has anyone close to you tried to hurt you or harm you physically, emotionally or mentally? (yes-no)

Has anyone in your whānau taken your belongings without your consent? (yes-no)

Cultural Identity
How important is your hapū to your wellbeing? (not at all important-extremely important)

How important is your iwi to your wellbeing? (not at all important-extremely important)

Sense of Purpose
I have a sense of direction and purpose in life. (strongly disagree to strongly agree)

I set goals for myself. (strongly disagree to strongly agree)

I enjoy making plans for the future and working to make them a reality. (strongly disagree to strongly agree)

Health Service Use and Knowledge
How much knowledge would you say you have about services that are available to you? (very much-not at all)

How much do health and social services help you with your needs? (very much-not at all)

How likely are you to get help with a housing, health, and social need if you need it? (extremely likely-not at all)

Housing Problems
How much of a problem is the quality of your house in terms of being damp, cold, mouldy, leaky or in need of repairs? (not a problem-a big problem)

How much of a problem is the financial aspects of your housing situation such as affording to live in a stable housing situation? (not a problem-a big problem)

How much of a problem is your housing situation to have autonomy, such as have room or space of your own? (not a problem-a big problem)

Historical Trauma
How often to you think about:

Loss of connection to hapū and iwi (several times a day-yearly/special times)

Loss of our language (several times a day-yearly/special times)

Losing our traditional cultural ways (tikanga) (several times a day-yearly/special times)

Loss of mana motuhake (several times a day-yearly/special times)

Understanding Tikanga (Cultural Protocols)
How well do you understand your tikanga? (not at all-completely)

Missing Pleasure of Being with Whānau (Extended Family)
I miss the pleasure of being with whānau often. (strongly disagree to strongly agree)

Needing More Help with Daily Tasks
Thinking about the past 4 weeks, could you have used more help with daily tasks than you received? (always-never)

Needing More Emotional Support
Thinking about the past 4 weeks, could you have used more emotional support than you received? (always-never)

Perceived Autonomy
On a scale of 0 being no control to 10 complete control, how much control do you have in your life?

Trouble Paying Bills
In the last 12 months, have you not paid electric, water bill on time because of a shortage of money? (not at all-more than once)

*Recoded item for scale
